# Efficacy and Safety of a Novel Oral Rehydration Solution (ORS) in Managing Diarrhea and Dehydration: A Randomized Study in Indian Patients

**DOI:** 10.7759/cureus.103248

**Published:** 2026-02-08

**Authors:** Bridreth Khokhare, Nutan Agrawal, Mohammed Siddique, Apoorv Gupta, Komal Pathak, Navodit Tiwari, Shubham Sahu, Uday Kumar

**Affiliations:** 1 Department of Research and Development, Mystical Biotech Pvt. Ltd., Bengaluru, IND; 2 Department of Medicine, Maharani Laxmi Bai (MLB) Medical College, Jhansi, IND

**Keywords:** acute diarrhea, dehydration, diarrhea, gastroenteritis, oral rehydration solution, probiotics

## Abstract

Background: Acute diarrhea is a leading cause of dehydration-related morbidity. Oral rehydration solution (ORS) remains the cornerstone of treatment, while probiotics have shown additional gut health benefits. This study evaluated the efficacy and safety of a novel probiotic-enriched ORS compared with a standard ORS in Indian patients with acute diarrhea.

Methods: In this randomized, parallel-group study, patients with diarrhea were enrolled and assigned to receive either novel ORS or traditional ORS. Treatment was administered for five days, followed by a four-day observation period and a safety follow-up call. Group 1 received the novel ORS (200 mL/day) for five days, and Group 2 received traditional ORS (200 mL/day) for a similar duration. Efficacy outcomes measured included resolution of diarrhea, time to recovery, patient-reported satisfaction and preference, and Clinical Global Impression-Improvement (CGI-I) score.

Results: In this randomized, parallel-group study, 60 patients with diarrhea were enrolled and assigned (3:1) to receive either novel ORS (n = 45) or traditional ORS (n = 15). Both groups showed clinical improvement; however, the novel ORS group demonstrated faster resolution. By visit 3, the mean diarrhea episodes were 0.4 (0.62) in the novel ORS group, while 0.9 (0.86) in the traditional group. Higher satisfaction was reported in the novel ORS group (42.2% "very satisfied") along with quicker improvement on the CGI-I score (mean score 1.3 vs. 1.9; p = 0.0120).

Conclusion: The novel ORS containing probiotics showed faster symptom resolution, higher patient satisfaction, and comparable safety, supporting its potential as a preferred treatment option for diarrhea management.

## Introduction

Acute diarrhea remains a major public health issue worldwide. According to the World Health Organization (WHO), it is characterized by the passage of three or more loose or watery stools within 24 hours [1]. The condition most often arises from gastrointestinal tract infections caused by bacteria, viruses, or parasites [1]. In developing nations such as India, diarrhea-related dehydration remains one of the leading contributors to illness and death [2].

Probiotics have shown promise in alleviating acute diarrhea by shortening its duration and severity; however, these beneficial effects are strain-specific and depend on the probiotic species, dose, and clinical context [3]. These are live microorganisms, mainly beneficial bacteria, that resemble the natural flora of the human intestine [4], commonly known as “good” or “friendly” bacteria. Probiotics help restore a healthy gut ecosystem, particularly after it has been disrupted by disease or medication [5].

Oral rehydration solution (ORS) remains the mainstay of treatment for acute diarrhea and associated dehydration worldwide [6]. While mild cases often resolve without medical intervention, severe diarrhea can result in extensive fluid and electrolyte loss, causing dehydration that may require hospitalization. The principal therapeutic goals are to maintain hydration, replace lost electrolytes, and accelerate recovery [7].

Initial management focuses on adequate fluid intake, appropriate nutrition, and dietary adjustment as needed [7]. ORS, a carefully formulated mixture of clean water, glucose, and electrolytes such as sodium, facilitates rapid fluid absorption through the intestinal wall [8]. Endorsed by WHO, ORS helps restore electrolyte balance and prevents hypovolemia during diarrheal episodes [9]. Since its introduction in the 1970s, ORS has saved countless lives, yet it does not directly target intestinal microflora imbalance, pathogen elimination, or mucosal recovery [10]. These limitations have prompted interest in supplementing ORS with probiotics.

The term "probiotics" was first introduced by Lilly and Stillwell [11] and later refined by Parker in 1974 [12] to emphasize their beneficial role on host health. In acute infectious diarrhea, probiotics may inhibit disease-causing organisms, enhance mucosal immunity, and reduce gut lining inflammation [5,13]. Different probiotic strains, administered alone or in combination, have demonstrated potential benefit in a variety of gastrointestinal conditions [5,10,14-18]. They are widely regarded as safe for human use, with only mild and self-limiting side effects such as bloating or flatulence [19].

The concept of combining probiotics with ORS has therefore gained attention. Such a formulation is expected to be affordable, safe, and stable during prolonged storage while also reducing stool output and shortening illness duration. The proposed mechanism for oral rehydration therapy involves enhanced water absorption through sodium-glucose co-transporters in the intestinal villi, primarily attributable to the hypo-osmolar composition of the solution [20], whereas probiotics exert their effects through biological mechanisms such as modulation of gut microbiota, enhancement of mucosal barrier function, and immune regulation [21]. A combined ORS-probiotic preparation could also increase patient compliance and reduce dependence on nonspecific antidiarrheal drugs. Although ORS remains the cornerstone of diarrheal management [22], variations in its formulation and quality among commercial preparations exist. Evidence indicates that co-administration of specific probiotics with ORS can significantly reduce the severity and duration of diarrhea, particularly in viral or parasitic infections [23].

Based on these considerations, the present prospective, randomized, comparative study was undertaken to evaluate the safety and efficacy of a novel ORS formulation containing probiotics versus a conventional ORS in Indian adults with acute diarrhea to prevent dehydration and improve clinical outcomes.

## Materials and methods

Study design

This was a nine-day-long, prospective, randomized, comparative, parallel-group study designed to evaluate the safety and efficacy of a novel ORS compared with a traditional marketed ORS in participants diagnosed with acute diarrhea. The study was conducted at a single clinical site in India between June 10, 2024, and July 24, 2024.

Participants in the investigational group received the novel ORS at a dosage of two sachets (4.6 g) per day, while those in the comparator group received one sachet (4.4 g) per day. Each sachet was diluted in 200 mL of drinking water and administered for five consecutive days. Telephonic follow-ups were performed on Days 3 and 5, and a final in-person assessment was conducted four days after treatment completion to evaluate safety and efficacy outcomes.

All enrolled participants, regardless of treatment allocation, received the standard of care for diarrhea management, encompassing dietary guidance and supportive therapy as required. The study was prospectively registered on the Clinical Trials Registry of India (CTRI/2024/05/067859).

Ethical considerations

This study was conducted in accordance with the ethical principles of the Declaration of Helsinki, Good Clinical Practice (GCP), the International Council on Harmonization (ICH) guidelines, and the New Drugs and Clinical Trials Rules, 2019 (Gazette notification G.S.R.227 (E), dated 19.03.2019). Approval from the Institutional Ethics Committee of Maharani Laxmi Bai Medical College and Associated Hospital (approval no.: ECR/1393/Inst/UP/2020) was obtained before study initiation.

Study participants

Eligible participants were male and female adults of Indian origin, aged 18-60 years, who were clinically diagnosed with acute diarrhea, defined as the passage of three or more loose or watery stools within a 24-hour period, with a symptom duration of less than 72 hours at the time of enrollment. Only participants who provided written informed consent and agreed to comply with study procedures were enrolled.

Participants with chronic diarrhea, presence of severe dehydration requiring immediate intravenous fluid replacement, known hypersensitivity to any component of either ORS, history of significant gastrointestinal disorders (e.g., inflammatory bowel disease, irritable bowel syndrome), and those who used antibiotics or any anti-diarrheal medication two weeks before study enrollment were excluded from the study. Individuals with a history of use of pre- or probiotics one week before study enrollment, who participated in another clinical trial within 30 days before study enrollment, and with underlying severe medical conditions (e.g., renal, cardiac, hepatic) that could affect hydration status or complicate assessment of study outcomes were excluded. Furthermore, subjects who used medications that could interfere with the study outcomes (e.g., antidiarrheal drugs) or had any other medical condition that would compromise the safety or compliance of the subject or the quality of the data were also excluded. Pregnant or breastfeeding women, as well as those planning to become pregnant during the study period, were excluded from participation.

Randomization and blinding

Once eligibility was confirmed, participants were randomized in a 3:1 ratio (novel ORS : traditional ORS) using a randomization schedule generated by an independent biostatistician to minimize allocation bias. Given the nature of the interventions, the study followed an open-label design, and thus blinding of investigators and participants was not feasible.

Interventions and follow-up

Participants in the investigational arm received the novel ORS formulation twice daily for five days, while the control group received the marketed ORS once daily for the same duration. Both products were administered orally after reconstitution in water.

Clinical evaluations were performed at baseline, on Day 3, Day 5 (end of treatment), and Day 9 (post-treatment). The telephonic follow-ups focused on assessing treatment adherence, symptom improvement, and any adverse effects.

Sample size estimation

Sample size was calculated to demonstrate non-inferiority between the test and comparator groups at a two-sided 5% significance level and 80% power, allowing for 10% dropouts, with allocation in a 3:1 ratio (45:15). The sample size estimation for non-inferiority testing was performed using standard formulas described by Chow et al. [24].

Study outcomes

Primary Outcomes

Primary endpoints included the proportion of participants achieving resolution of dehydration and the time taken for complete resolution of diarrhea symptoms. These outcomes were assessed at three time points: Visit 2 (Day 3), Visit 3 (Day 5), and Visit 4 (Day 9) (four days after treatment completion). Participant-reported satisfaction with ORS treatment was also collected to assess product preference.

Secondary Outcomes

Secondary assessments included investigator evaluations of treatment effectiveness and patient preference using the Clinical Global Impression-Improvement (CGI-I) scale [25,26].

Safety assessments

Safety evaluation was conducted from the time informed consent was obtained until study completion or until the resolution of any adverse event (AE) or serious adverse event (SAE), whichever occurred later. Safety monitoring included vital signs, physical examinations, and clinical laboratory investigations at scheduled visits. All AEs were documented, including details on onset, duration, severity, seriousness, relationship to the study product, treatment provided, and outcome.

Statistical analysis

Efficacy and safety analyses were conducted on intent-to-treat (ITT) and safety populations. Continuous variables were summarized as mean (standard deviation (SD)), while categorical variables were expressed as frequency and percentage.

Safety data were analyzed descriptively by visit and treatment group. AEs and SAEs were coded using MedDRA version 24.0, categorized by System Organ Class (SOC) and Preferred Term (PT), and summarized by the number and percentage of affected participants and total number of events.

AEs were compared between treatment groups. Descriptive summaries were also generated for clinical laboratory results and vital signs. All statistical analyses were performed using IBM SPSS Statistics for Windows, Version 26 (Released 2019; IBM Corp., Armonk, New York), with a significance threshold of p < 0.05.

## Results

**Figure 1:**
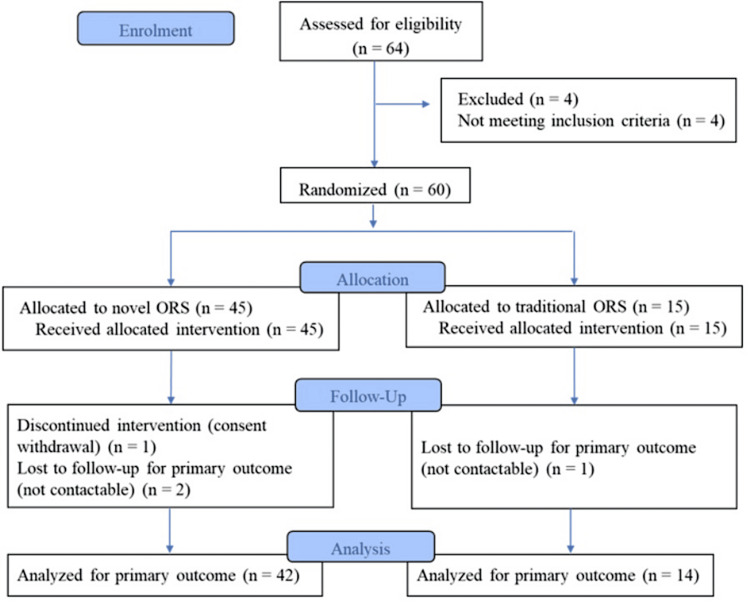
CONSORT flow chart CONSORT: Consolidated Standards of Reporting Trials

A total of 64 participants were screened according to the predefined eligibility criteria. Of these, four participants did not meet the inclusion criteria, leaving 60 eligible participants who were enrolled and randomized into the study. Data from all 60 participants were included in the final analysis (Figure 1).

Demographic and baseline characteristics

Demographic and baseline characteristics were comparable between the two treatment groups (Table 1).

**Table 1:** Demographic characteristics Data presented as n (%), unless otherwise specified. ORS: oral rehydration solution; BMI: body mass index; SD: standard deviation; T2DM: type 2 diabetes mellitus

Parameters	Novel ORS Group (N = 45)	Traditional ORS (N = 15)
Age (years), mean (SD)	34.4 (9.33)	35.6 (10.72)
BMI (kg/m^2^), mean (SD)	23.6 (2.01)	23.3 (2.19)
Sex		
Male	30 (66.7)	13 (86.7)
Female	15 (33.3)	2 (13.3)
Medical history		
Hypertension	3 (6.7)	0
T2DM	0	1 (6.7)
Gastrointestinal Signs/Symptoms		
Vomiting	26 (57.8)	9 (60.0)
Abdominal pain	15 (33.3)	5 (33.3)
Nausea	1 (2.2)	0 (0.0%)
Other Signs/Symptoms		
Headache	4 (8.9)	1(6.7)
Fever	5 (11.1)	6 (40.0)
Concomitant Medications	32 (71.1)	13 (86.7)
Ondansetron	9 (20.0)	3 (20.0)
Mefenamic acid	7 (15.6)	2 (13.3)
Pantoprazole	6 (13.3)	1 (6.7)
Paracetamol	4 (8.9)	5 (33.3)
Omeprazole	4 (8.9)	1 (6.7)
Telmisartan	2 (4.4)	0
Dicyclomine	1 (2.2)	0

The mean (SD) age was 34.4 (9.33) years and 35.6 (10.72) years in the novel ORS group and traditional ORS group, respectively. The novel ORS group had 30 (66.7%) males and 15 (33.3%) females, while the traditional ORS group had a higher proportion of males (n = 13; 86.7%) and fewer females (n = 2; 13.3%). Gastrointestinal complaints were the most frequently reported baseline symptoms. Abdominal pain was reported in 15 (33.3%) of participants in each group, while nausea was infrequent, observed in only one (2.2%) of those receiving the novel ORS. Vomiting was the most common presenting symptom, reported in 26 (57.8%) of participants in the novel ORS group and nine (60.0%) in the traditional ORS group. A history of fever was more frequently reported in the traditional ORS group (n = 6; 40.0%) compared with the novel ORS group (n= 5; 11.1%). The use of supportive medications was comparable between groups; however, the traditional ORS group showed a higher overall frequency of concomitant medication use, particularly paracetamol (n = 4; 8.9% in the novel ORS group vs. n = 5; 33.3% in the traditional ORS group).

Primary outcome measure

**Figure 2:**
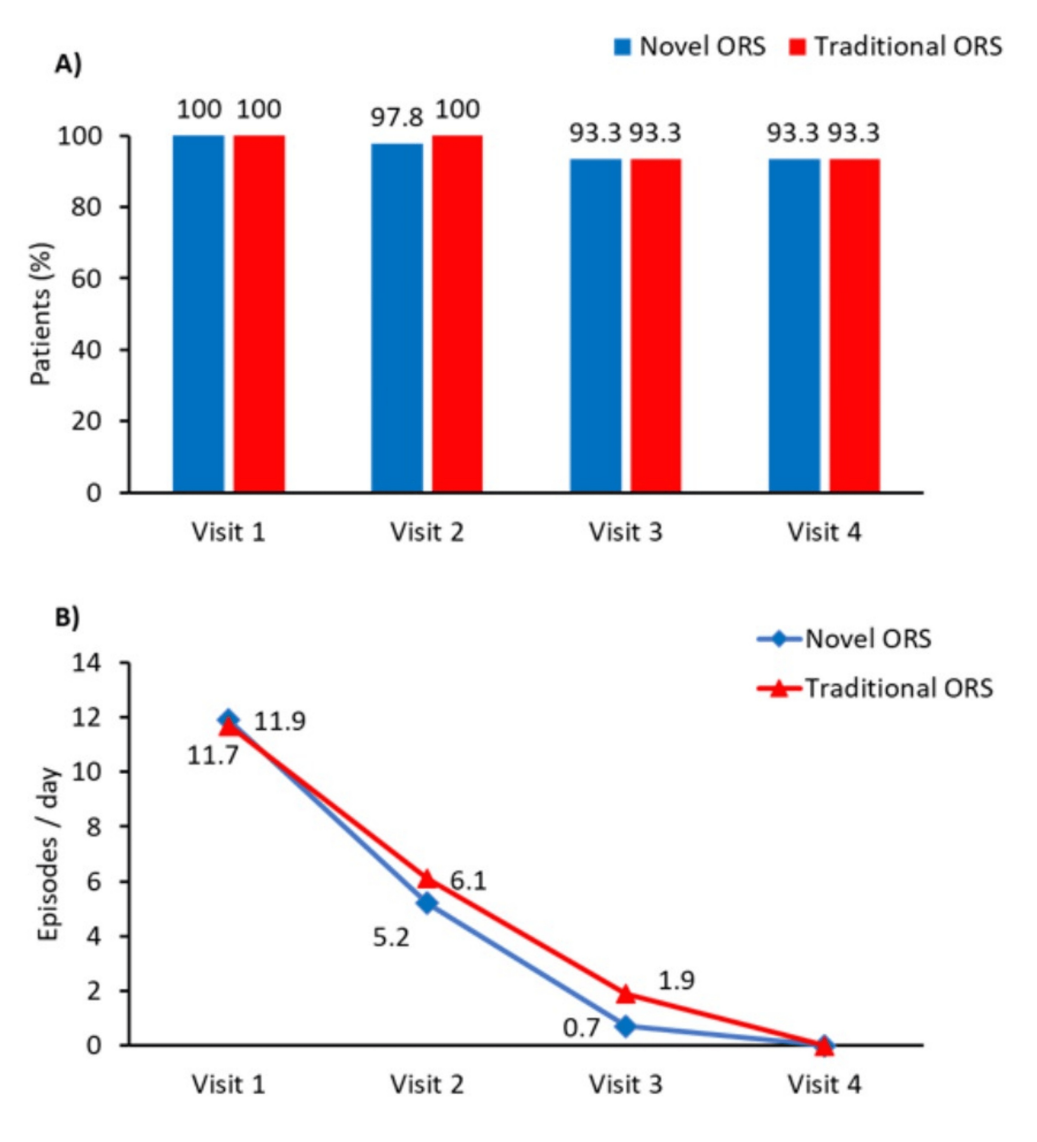
Primary outcomes (A) Proportion of participants achieving resolution of dehydration; (B) time taken for complete resolution of diarrhea symptoms. ORS: oral rehydration solution

By Visit 2, dehydration had resolved in 44 (97.8%) of participants in the novel ORS group and 15 (100%) in the traditional ORS group. By Visit 3, recovery was maintained in 93.3% of both groups, with no additional improvement noted at Visit 4, where recovery remained at 93.3% (Figure 2A). The mean duration of diarrhea at Visit 1 was comparable between groups (11.9 days in the novel ORS group vs. 11.7 days in the traditional ORS group). By Visit 3, the mean duration had markedly decreased to 0.7 days with the novel ORS and 1.9 days with the traditional ORS, indicating faster symptom resolution in participants receiving the novel ORS formulation (Figure 2B).

**Figure 3:**
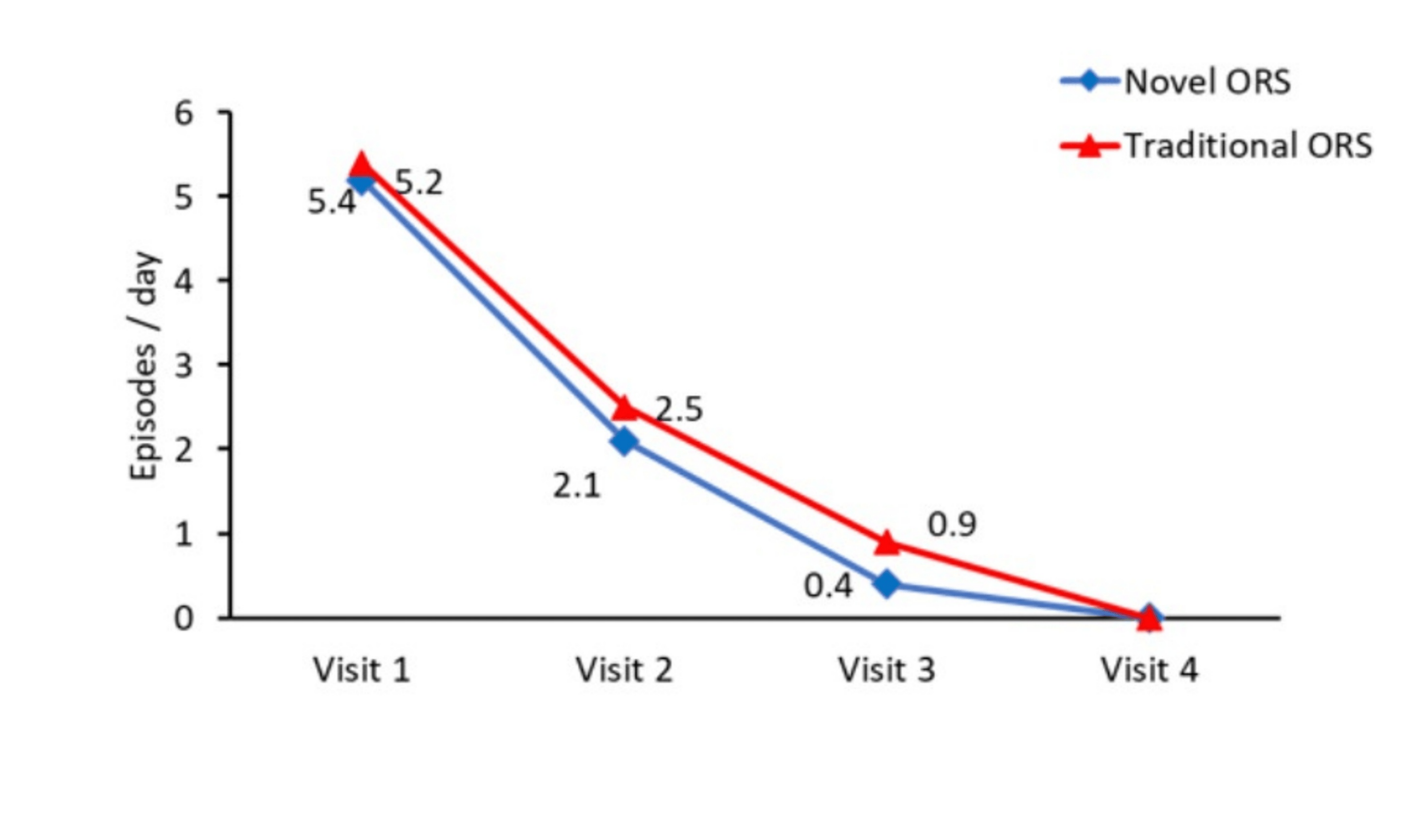
Number of episodes of diarrhea (per day) ORS: oral rehydration solution

By Visit 2, both groups showed significant improvement, with the mean number of diarrhea episodes reduced to 2.1 (0.74) in the novel ORS group and 2.5 (0.74) in the traditional ORS group. By Visit 3, further improvement was observed, with episodes decreasing to 0.4 (0.62) in the novel ORS group and 0.9 (0.86) in the traditional ORS group. Diarrhea was completely resolved in both groups by Visit 4 (Figure 3, Supplemental material 1).

The novel ORS group also reported higher satisfaction, with 19 (42.2%) of participants indicating they were “very satisfied" and 15 (33.3%) reporting they were “satisfied.” In contrast, a large majority (n = 14; 93.3%) of participants in the traditional ORS group expressed uncertainty about their satisfaction level. Similarly, treatment preference favored the novel ORS formulation. Among those receiving the novel ORS, 19 (42.2%) expressed a strong preference and 15 (33.3%) agreed with their preference for the formulation, whereas 13 (86.7%) of participants in the traditional ORS group were “not sure” and only one (6.7%) indicated a preference for the traditional product (Table 2).

**Table 2:** Satisfaction and preference - safety population (N=60) Data presented as n (%). ORS: oral rehydration solution

Parameter	Novel ORS Group (N = 45) n (%)	Traditional ORS group (N = 15) n (%)
How satisfied is the subject with the novel ORS when compared to traditional ORS		
Very Satisfied	19 (42.2)	0
Satisfied	15 (33.3)	0
Neither satisfied nor dissatisfied	8 (17.8)	14 (93.3)
The subject will prefer the novel ORS over traditional ORS		
Strongly Agree	19 (42.2)	0
Agree	15 (33.3)	1 (6.7)
Not Sure	8 (17.8)	13 (86.7)

Secondary outcome measure

Investigator assessments using the CGI-I scale showed a similar trend observed in clinical recovery (Table 3).

**Table 3:** CGI assessments safety population The p-value within the group was calculated using the Wilcoxon signed-rank test, and the p-value between the groups was calculated using the Mann-Whitney U test at the 5% significance level. ORS: oral rehydration solution; CGI: Clinical Global Impression-Improvement

Visit	Parameter	Novel ORS Group (N = 45)	Traditional ORS (N = 15)
CGI assessment			
Visit 1	Minimally worse	26 (57.8)	8 (53.3)
	Much worse	18 (40.0)	6 (40.0)
	Very much worse	1 (2.2)	1 (6.7)
Visit 2	Minimally worse	4 (8.9)	-
	Much worse	27 (60.0)	5 (33.3)
	Very much worse	13 (28.9)	10 (66.7)
Visit 3	Minimally worse	28 (62.2)	4 (26.7)
	Much worse	14 (31.1)	9 (60.0)
	Very much worse		1 (6.7)
CGI score			
Visit 1	n	44	14
	Mean (SD)	5.4 (0.5)	5.4 (0.51)
	p-value between groups	0.9072	
Visit 2	n	44	15
	Mean (SD)	2.2 (0.59)	2.7 (0.49)
	Mean change (SD)	-3.2 (0.57)	-2.8 (0.70)
	p-value within groups	<0.0001	<0.0001
	p-value between groups	0.0118	
Visit 3	n	42	14
	Mean (SD)	1.3 (0.48)	1.9 (0.77)
	n, mean change (SD)	41, -4.1 (0.66)	13, -3.7 (0.75)
	p-value within groups	<0.0001	<0.0001
	p-value between groups	0.0120	

The novel ORS group showed a more rapid and greater degree of improvement, with a lower mean CGI-I score at Visit 3 compared with the traditional ORS group (1.3 vs. 1.9; p = 0.0120). The mean CGI score also exhibited significant improvement from baseline to subsequent visits in both groups (p < 0.0001); however, the novel ORS group demonstrated significantly greater improvement compared to the traditional ORS group on both Visit 2 (p = 0.0118) and Visit 3 (p = 0.0120). These findings suggest a more favorable and faster clinical response with the novel ORS formulation compared to the traditional product.

Safety assessment

Both formulations showed an excellent safety profile, with no serious AEs reported throughout the study. The overall incidence of AEs was 3 (6.7%) in the novel ORS group and 2 (13.3%) in the traditional ORS group (Table 4).

**Table 4:** Safety assessment Data presented as n (%). ORS: oral rehydration solution

Parameter/Visit	Severity	Relationship	Novel ORS Group (N = 45)	Traditional ORS (N = 15)
Number of subjects with at least one overall adverse event	-	-	3 (6.7)	2 (13.3)
Pyrexia	Mild	Unlikely	2 (4.4)	0
Back pain	Mild	Unlikely	0	1 (6.7)
Headache	Mild	Unlikely	0	1 (6.7)
Sneezing	Mild	Unlikely	1 (2.2)	0

All reported AEs were mild in intensity and assessed as unlikely related to the study interventions. In the novel ORS group, the most common AEs reported were mild pyrexia (n = 2; 4.4%) and sneezing (n = 1; 2.2%). In the traditional ORS group, AEs primarily involved the musculoskeletal (n = 1; 6.7%) and nervous system (n = 1; 6.7%) categories. No clinically meaningful changes were observed in vital signs, physical examinations, or laboratory parameters across study visits. A minimal, non-clinically significant decrease in heart rate was observed by Visit 3.

## Discussion

This study evaluated the efficacy and safety of a novel ORS compared with the traditionally available ORS in adults with acute diarrhea. The novel formulation demonstrated faster resolution of diarrhea, greater participant satisfaction, and a similar safety profile.

Diarrhea and dehydration are closely interrelated; diarrheal episodes cause excessive loss of fluids and electrolytes (like sodium, potassium, and chloride), which can rapidly lead to dehydration if not corrected [8]. The severity of dehydration correlates directly with the amount and the duration of fluid loss [27]. The novel ORS tested in this study was formulated with an optimized salt-to-glucose ratio that facilitates intestinal absorption via the sodium-glucose cotransport mechanism, enabling effective rehydration even during active diarrheal episodes. This improved fluid retention and restoration of electrolyte balance facilitated symptom resolution and promoted recovery. As the frequency and volume of stool decreased, ongoing fluid loss was minimized, further accelerating rehydration.

Our study showed that by Visit 3, the mean number of diarrheal episodes had reduced to 0.4 in the novel ORS group compared with 0.9 in the traditional ORS group, and diarrhea had completely resolved by Visit 4. These findings support the fundamental principle that effective ORS therapy simultaneously corrects dehydration and aids diarrheal recovery, consistent with WHO guidelines and prior clinical evidence [28].

Probiotics can help with diarrhea by addressing microflora imbalance, pathogen clearance, and environmental enteric dysfunction, which ORS alone cannot do [10]. This finding was supported by the present study, in which participants receiving the novel ORS reported significantly greater preference and satisfaction compared to those in the traditional ORS group. Previous studies have also demonstrated that probiotics may help reduce the duration of diarrhea and decrease stool frequency [16-18]. Also, systematic reviews of the use of probiotics as an adjunct therapy to ORS have shown that probiotics shorten the duration of diarrhea and reduce stool frequency [14,15,23]. However, the observation remains inconclusive due to the small number of participants in the few trials. In this study, the incorporation of probiotics into the ORS formulation was associated with a reduced duration of diarrhea.

ORS is generally safe and tolerable in patients with diarrhea; however, stomach discomfort, fluid overload, hyperkalemia, or hyponatremia may occur. However, in the present study, no such AEs were observed. Also, patients' acceptance of the novel ORS and satisfaction were positive signs of the product’s tolerability.

Strengths and limitations

Among the strengths of our study are its prospective randomized design, clearly defined efficacy and safety outcomes, and systematic safety assessment conducted under ethical oversight. The study evaluated both objective clinical endpoints and patient-reported outcomes in a clinically relevant adult Indian population, which is underrepresented in ORS and probiotic research.

Nevertheless, the study has a few limitations. First, the single-center and small sample size may limit the statistical power and generalizability of the findings. Second, the open-label design and 3:1 allocation ratio introduce potential bias, especially in subjective endpoints such as treatment preference. Third, while the novel ORS formulation appears effective, the mechanistic contribution of the probiotic component was not directly assessed (e.g., by microbiome analysis or pathogenic load). Fourth, follow-up was limited to nine days; long-term outcomes, recurrence rates, or cost-effectiveness were not evaluated. In addition, unequal dosing between treatment arms and the use of supportive therapies may have resulted in residual confounding. Finally, while our results suggest benefit in adults, the majority of existing probiotic/ORS adjunct data are from pediatric populations, making direct comparisons difficult.

## Conclusions

The novel ORS containing probiotics was more effective and preferred by participants compared with the traditional ORS. Participants receiving the novel formulation showed faster resolution of diarrheal symptoms, greater clinical improvement, and higher satisfaction, while both treatments exhibited comparable safety and tolerability. These findings highlight the potential of the probiotic-enriched ORS as an effective, well-tolerated, and patient-preferred option for managing acute diarrhea. Its use may be particularly beneficial in resource-limited settings where diarrhea-related dehydration remains a major public health concern.

## Appendices

Supplemental material 1 

**Table 5:** Diarrhea assessment ORS: oral rehydration solution; SD: standard deviation; CI: confidence interval

Parameters	Novel ORS Group (N = 45)	Traditional ORS (N = 15)
Is the diarrhea symptom assessment done? n (%)		
Visit 1	45 (100.0)	15 (100.0)
Visit 2	44 (97.8)	15 (100.0)
Visit 3	42 (93.3)	14 (93.3)
Visit 4	42 (93.3)	14 (93.3)
Total duration of a diarrhea episode (per day), mean (SD); 95% CI		
Visit 1	11.9 (2.88); 11.0, 12.8	11.7 (3.67); 9.7, 13.8
Visit 2	5.2 (2.13); 4.6, 5.9	6.1 (1.91); 5.0, 7.1
Visit 3	0.7 (1.31); 0.3, 1.1	1.9 (2.23); 0.6, 3.2
Visit 4	0	0
No. of episodes of diarrhea (per day), mean (SD); 95% CI		
Visit 1	5.2 (0.88); 5.0, 5.5	5.4 (0.83); 4.9, 5.9
Visit 2	2.1 (0.74); 1.9, 2.3	2.5 (0.74); 2.1, 2.9
Visit 3	0.4 (0.62); 0.2, 0.5	0.9 (0.86); 0.4, 1.4
Visit 4	0	0
